# Altered Olfactory Processing of Stress-Related Body Odors and Artificial Odors in Patients with Panic Disorder

**DOI:** 10.1371/journal.pone.0074655

**Published:** 2013-09-24

**Authors:** Gloria-Beatrice Wintermann, Markus Donix, Peter Joraschky, Johannes Gerber, Katja Petrowski

**Affiliations:** 1 Department of Psychotherapy and Psychosomatic Medicine, Dresden University of Technology, Medical School, Dresden, Germany; 2 Department of Psychiatry and Psychotherapy, Universitätsklinikum Carl Gustav Carus, Dresden, Germany; 3 Department of Neuroradiology, Dresden University of Technology, Medical School, Dresden, Germany; The University of Melbourne, Australia

## Abstract

**Background:**

Patients with Panic Disorder (PD) direct their attention towards potential threat, followed by panic attacks, and increased sweat production. Onés own anxiety sweat odor influences the attentional focus, and discrimination of threat or non-threat. Since olfactory projection areas overlap with neuronal areas of a panic-specific fear network, the present study investigated the neuronal processing of odors in general and of stress-related sweat odors in particular in patients with PD.

**Methods:**

A sample of 13 patients with PD with/ without agoraphobia and 13 age- and gender-matched healthy controls underwent an fMRI investigation during olfactory stimulation with their stress-related sweat odors (TSST, ergometry) as well as artificial odors (peach, artificial sweat) as non-fearful non-body odors.

**Principal Findings:**

The two groups did not differ with respect to their olfactory identification ability. Independent of the kind of odor, the patients with PD showed activations in fronto-cortical areas in contrast to the healthy controls who showed activations in olfaction-related areas such as the amygdalae and the hippocampus. For artificial odors, the patients with PD showed a decreased neuronal activation of the thalamus, the posterior cingulate cortex and the anterior cingulate cortex. Under the presentation of sweat odor caused by ergometric exercise, the patients with PD showed an increased activation in the superior temporal gyrus, the supramarginal gyrus, and the cingulate cortex which was positively correlated with the severity of the psychopathology. For the sweat odor from the anxiety condition, the patients with PD showed an increased activation in the gyrus frontalis inferior, which was positively correlated with the severity of the psychopathology.

**Conclusions:**

The results suggest altered neuronal processing of olfactory stimuli in PD. Both artificial odors and stress-related body odors activate specific parts of a fear-network which is associated with an increased severity of the psychopathology.

## Introduction

Panic disorder (PD) is characterized by sudden bursts of panic attacks accompanied by heart palpitations, dizziness, trembling, and sweating [1], [2]. Until now, the exact pathophysiological mechanism which triggers panic attacks has not been understood completely [3]. The neuroanatomical model of PD suggests a general disturbance of information processing with an attentional bias for threat-related stimuli from the environment [4], [5]. Besides visuospatial stimuli, auditory stimuli or cognitive misinterpretation, recent findings suggest olfactory stimuli as relevant triggers or at least catalysts for panic attacks [6], [7]. During the state of anxiety, an individuaĺs own anxiety sweat conveys information about the possible threat of a situation. The release of chemosensory anxiety signals influences the attentional focus on vigilance and facilitates the discrimination between threat and non-threat [8], [9], [10], [11], [12]. Thus, the perception of onés own anxiety sweat odor might function as a chemosensory feedback system which might trigger or catalyze panic attacks in patients with PD [10], [13].

Since anxiety sweat is processed much faster than other olfactory stimuli [14], [15], [16], specific brain areas relevant for the representation of the social and emotional significance of stimuli (amygdalae, cingulate cortex) and attentional regulation (thalamus, parietal cortex) are involved [9], [17], [18], [19], [20], [21], [22]. Moreover, the neuronal processing of anxiety sweat is localized in areas associated with the regulation of empathetic feelings (insula, precuneus, cingulate cortex), attentional control (dorsomedial prefrontal cortex, dmPFC), and emotional control (cerebellum, vermis) [20]. These areas which are mainly involved in the regulation of emotions, memory, and attentional control have been shown to be altered in patients with PD [5], [7], [23], [24], [25], [26], [27], [28], [29]. Most fMRI studies showed the involvement of the amygdalae as central fear structure with increased activation [5], [27], [28], [30] under the stimulation with threat-related stimuli, emotional conflict detection (e.g. emotional Stroop task, [5]; face-word-pairs, [27]), and the occurrence of panic [7] or decreased activation under the presentation of fearful face-stimuli [25]. Also, studies could show the involvement of the anterior/posterior cingulate cortex with either decreased activation under the stimulation with fearful stimuli [25] or increased activation under the presentation of happy face stimuli [26], neutral face stimuli [25], threat-related words [24], and under the imagery of severe anxiety [23]. Increased activation of the hippocampus, the orbitofrontal cortex, and the inferior frontal cortex could be shown in patients with PD when compared with healthy controls under the imagery of severe vs. neutral anxiety conditions [23]. Furthermore, enhanced activation of the inferior frontal gyrus has been demonstrated using the presentation of agoraphobia-specific pictures [30] and differential conditioning [31].

Alterations within three major neuronal networks have been associated with the aetiopathogenesis of PD [4], [29]. Recent findings showed an increased resting state functional connectivity of the amygdalae with the posterior cingulate cortex, the precuneus, and the occipital cortex in the limbic network [29]. Aberrant connectivity of the dorsal anterior cingulate cortex with the pre- and postcentral gyrus has been found in a so-called salience network [29]. According to the neuroanatomical model of PD, an abnormally sensitive fear-network is associated with the occurrence of panic [4], [32]. This model postulates an aberrant stimulus processing from the cortex and the brainstem, which leads to an excitatory input to the amygdalae that evaluates sensory input in terms of potential threat. The amygdalae are reciprocally connected with parts of the brainstem, the sensory thalamus, the insula, the medial prefrontal cortex, and the cingulate cortex. According to this model, the amygdalae can be directly excited via input from the medial prefrontal and primary somatosensory cortex, which has been associated with the misinterpretation of somatic information [4], [32]. Via efferent projections to the medial hypothalamus and the nuclei of the brainstem, the panic-type behavioural, endocrine, and autonomic responses are released. Above all, afferent projections from the hippocampus can directly stimulate the amygdalae [32]. Thus, the neuronal areas which are functionally altered in patients with PD (limbic or fear network and salience network) overlap with neuronal areas of the olfactory system and the neuronal areas involved in the processing of anxiety sweat odor [4], [5], [23], [24], [29], [32]. However, studies in patients with PD considering the functional neuronal representation of olfactory stimuli, especially fear-related olfactory stimuli, are lacking. To which extent fear-related olfactory stimuli can trigger a panic-specific fear network in PD is unknown. Therefore, in the present study onés own anxiety sweat odor was presented during fMRI and contrasted with a stress-related sweat control odor (bicycle ergometry) and non-fear-related artificial odors (peach odor, artificial sweat odor). Due to the overlap of the panic-specific fear network with the projection areas of the olfactory system (e.g. amygdalae, orbitofrontal cortex, insula, hippocampus, thalamus) it can be assumed that the neuronal processing of odors in patients with PD may be altered, in general [4], [33], [34]. Furthermore, previous studies were able to show that especially fear-related odors activate neuronal areas where patients with PD show alterations which are likewise part of the limbic network (e.g. amygdalae, cingulate cortex, insula, precuneus, dmPFC/inferior frontal gyrus) [9], [20]. Since patients experiencing severe panic show an exaggerated activation of the sympathico-adrenomedullary system accompanied by enhanced sweating [35] it is assumed that a patients own anxiety sweat odor may be a potent stimulus for activating the panic-specific fear-network.

## Materials and Methods

### Ethics Statement

The present investigation was performed according to the Declaration of Helsinki on Biomedical Research Involving Human Subjects and was approved by the University of Dresden Medical Faculty Ethics Review Board (EK: 24022009). After description of the complete study protocol participants signed in a written informed consent.

### Participants

Participants for the study were recruited in an outpatient unit of the clinic for psychosomatic medicine and psychotherapy at the University Hospital of the Dresden University of Technology, Dresden, Germany from June 2009 to January 2011. The Structured Clinical Interview (SCID) [36], [37] for the Diagnostic and Statistical Manual of Mental Disorders (DSM-IV) was used to ascertain a diagnosis of panic disorder with agoraphobia [38]. The patients did not suffer from any other mental disease in their lifetime, and were free of any medication. Female participants were permitted to use oral contraceptives (see Table 1). The healthy individuals were recruited by public advertisements.

**Table 1:** Characteristics of patients with Panic Disorder (PD) (N = 13) and healthy controls (N = 13). Displayed are the means and standard deviations (S.D.) or percentages.

	Patients with PD	Controls (C)	*χ^2^/U*	*P*
Total, N	13	13		
Females, n (%)	6 (46.2)	6 (46.2)		
Males, n (%)	7 (53.9)	7 (53.9)	.000	1.000(†)
Age (years)	31.10 (13.01)	24.82 (4.52)	70.000	.457(U)
Cycle week	2.00 (1.22)	2.00 (.89)	14.000	.848(U)
Oral contraceptives, n (%)	2 (33.33)	1 (16.67)	.444	1.000(†)
BMI (kg/m^2^)	22.26 (2.40)	23.64 (3.28)	64.000	.293
Smokers, n (%)	6 (46.2)	2 (15.4)	2.889	.202(†)
Alcohol consumption, n (%)	8 (61.5%)	8 (61.5%)	.000	1.000(†)
Contraceptive pill, n (%)	2 (33.3)	1 (16.7)	.749	.545(†)
PAS	16.32 (12.91)	1.91 (4.66)	17.500	<.001***(U)
STAI-Trait	41.92 (9.64)	33.77 (7.54)	45.500	.045*(U)
ACQ	.95 (.52)	.54 (.30)	45.000	.042*(U)
BSQ	1.50 (.78)	1.10 (.58)	61.000	.227(U)
MI alone	.86 (.93)	.24 (.40)	43.500	.035*(U)
MI accompanied	.60 (.70)	.08 (.11)	33.000	.008**(U)
BDI	10.69 (4.61)	3.85 (3.69)	18.000	<.001***(U)
GSI (SCL), T-value	67.09 (9.55)	52.08 (10.42)	19.500	.003**(U)
STAI-TSST, pre/post	37.77 (9.68)/49.85 (12.59)	39.00 (10.19)/45.46 (6.16)	77.500/65.000	.719(U)/.316(U)
STAI-ergo, pre/post	37.50 (7.17)/39.15 (9.06)	34.54 (3.64)/34.46 (5.35)	68.000/72.000	.395(U)/.520(U)

*** p<.001, **p<.01; *p<.05; † Chi-square test; U =  Mann-Whitney U-Test; ACQ  =  Agoraphobic Cognitions Questionnaire [42]; BDI  =  Beck-Depression-Inventory [39]; BSQ  =  Body Sensations Questionnaire [42]; ergo  =  ergometry; GSI  =  General-Symptom-Index [40]; MI  =  Mobility Inventory [43]; PAS  =  Panic and Agoraphobia- Scale [41]; SCL  =  Symptom Check List [40]; STAI  =  State-Trait-Anxiety-Inventory [44].

For the evaluation of the depressive symptomatology, the Beck-Depression Inventory (BDI, [39]) was used. For the evaluation of psychological impairment, the Symptom-Check-List (SCL, [40]) was applied. The Panic and Agoraphobia-Scale (PAS, [41]) was used to assess the symptom severity for phobic anxiety. Moreover, to assess body-related anxiety, cognitions, and avoidance, the Body Sensations Questionnaire (BSQ, [42]), the Agoraphobic Cognitions Questionnaire (ACQ, [42]) and the Mobility Inventory (MI, [43]) were used. The extent of state and trait anxiety was assessed with the State-Trait-Anxiety-Inventory (STAI, [44]).

Of the N = 14 (seven females, seven males) patients participating in the present study, one had to be excluded because the patient broke off the fMRI examination. The remaining patient sample consisted of six females and seven males (mean age  = 31.10, SD  = 13.01).

Of the group of healthy controls (N = 17, eight females, nine males) four participants had to be excluded because of frontal signal loss in one patient, a cyst at the neuro-pituitary in another patient, and another two patients due to technical problems with the olfactometer.

The healthy controls (six females, seven males, mean age  = 24.82, SD  = 4.52) were matched to the patients by age and gender. There were no significant differences to the patients with respect to age, gender, use of contraceptive pills, menstrual cycle, smoking, body mass index (BMI), and alcohol consumption (see Table 1). The patients with PD had significantly higher scores on the PAS, the depression scale, and the general symptom index of the SCL as well as higher trait anxiety than healthy controls. The patients more often had anxious cognitions and more frequently avoided agoraphobic situations, alone or accompanied, than the healthy controls (Table 1).

All participants were right-handed and did not have any neurological diseases (e.g. epilepsy), acute or chronic nasal or respiratory diseases (e.g. rhinitis, sinusitis, hyposmia, anosmia), and were without medication with an impact on the olfactory system (e.g. ACE inhibitor, psychotropic drugs). Normal olfactory function was ascertained with the odor identification task using the ‘‘Sniffin’ Sticks’’ test kit [45], [46]. The two groups did not differ with respect to odor identification and all the participants showed normal olfactory function [T = .000, df = 24, p = 1.000; patients vs. controls, mean (SD): 10.54 (.88) vs. 10.54 (1.39)]. Above all, the two groups did not significantly differ in reference to what extent the four odors were correctly identified after each block [all p>.05].

### Sweat sampling

Sweat was sampled under standardized conditions using an odorless T-Shirt during two stress procedures: first, for the sampling of “anxiety sweat”, a psychosocial stress test of ten minutes duration consisting of a self-presentation task and an arithmetic task was realized (Trier Social Stress Test, TSST, [47]). Second, for the sampling of the participants’ own control sweat, a physical exercise condition (bicycle ergometry) of ten minutes duration with resistance of ten Watt, a minimum of 110 bpm and a maximum of 120 bpm was realized according to Pause et al. [48] and Prehn-Kristensen et al. [20]. Sweat odor from a moderately intense physical exercise condition was collected as sweat control odor. This situation is typically perceived to be emotionally neutral and associated with only increased physical but not anxious arousal [20], [48]. This point was proved by measuring the state anxiety with the state version of the State-Trait-Anxiety-Inventory (STAI) before and after the two stress conditions (TSST vs. ergometry) [44]. Above all, the affective dimension of experienced arousal was assessed using the Self-Assessment Manikin (SAM) as a nine-point pictorial rating scale before and after both stress conditions [49]. Both TSST and the ergometric exercise took place on two separate days with a mean interval of 15.08 days (SD = 19.41 days, minimum: 2 days, maximum: 80 days). Both tests lasted the same length of time and took place at the same time of day [9]. The room temperature was assessed with a stationary thermometer. The temperature was comparable between the groups and the conditions (TSST, bicycle ergometry) with no change throughout the test procedure. The patients were asked not to use any deodorants, perfumed shampoos, and soaps the day before and on the day of the sweat sampling. Moreover, the participants were asked not to eat meals with odor-intensive ingredients such as garlic, cabbage, or onions on both the day before and on the day of the sweat sampling. In addition, on the day of the sweat sampling the participants were asked to refrain from alcohol, coffee, and smoking, and to wash their armpits with an odorless soap shortly before the sweat sampling.

Both groups showed a significant increase in state anxiety over time (main effect of time: F(1, 24)  = 20.091, p<.001) with a significantly higher increase during the TSST than during the bicycle ergometry condition (interaction time x condition: F(1, 24)  = 13.032, p = .001). The two groups did not differ significantly with respect to state anxiety between conditions (interaction group x condition: F(1, 24)  = .583, p<.452). According to the SAM, both groups showed an increase in arousal (main effect of time: F(1, 24)  = 5.245, p = .031) with no significant difference between conditions (interaction time x condition: F(1, 24)  = .347, p = .561) and groups (interaction group x condition: F(1, 24)  = 1.215, p = .281).

The sweat samples were stored air-tight to be deep-frozen at −80° Celsius in the central laboratory at the University Hospital of the Dresden University of Technology, Dresden, Germany.

### Stimulus Presentation

We used a two-factorial design with the between subject factor, ‘‘group’’ (PD vs. controls), and the within subject factor ‘‘odor’’. The odors presented were either the participants’ own sweat odor (TSST sweat, ergometry sweat) or artificial odors (artificial sweat, peach).

Each subject participated in four sessions with two different body odors (TSST, bicycle ergometry) and two non-body odors. As non-body odors, the artificial olfactory stimuli “peach” [Frey und Lau GmbH, Henstedt-Ulzburg, Germany] and “artificial sweat” [Unilever, Port Sunlight, UK] were used. Each one of the four odors was presented birhinally in randomized order. The peach odor and the artificial sweat odor were dissolved in propylenglykol 1,2-propandiol (C_3_H_8_O_2_) 1:20. For the presentation of the sweat samples, the armpit area (10 by 10 cm) of the T-Shirts worn during the different stress conditions (TSST, bicycle ergometry) was cut out. The two swatches per sweat condition were placed in a wash bottle blown through by a constant air flow. Odors were presented intra-nasally (inner diameter of the TeflonTM tubing: 4 mm; length: 5 m) [50]. To avoid mechanical stimulation, the odor pulses were embedded in a constant flow of odorless, humidified air of 2.5 l/minute [50]. Stimulus pulses had a length of 2 seconds, the interval between the stimuli was 1 second. During the stimulus presentation, the participants were trained to breathe synchronously. Before the stimulus presentation, the participants were not supplied with any information about which stimulus would be presented. To ensure alertness, each subject had to rate the perceived intensity (0  = extremely low intensity; 10 =  extremely high intensity) and the hedonic quality (−5 =  extremely unpleasant; +5  = extremely pleasant) of each odor after each session.

### FMRI Protocol and Data Analysis

We used a 1.5T scanner (Siemens Magnetom Avanto, Erlangen, Sonata, Vision) for fMRI data acquisition. We utilized a blocked factorial design and presented the odors via an olfactometer during fMRI scanning to evoke the neural responses associated with the olfactory stimuli as had previously been described by Croy et al. [50].

For functional data, 96 volumes per session were acquired by means of a 33 axial-slice matrix 2D SE/EP sequence (TR: 2500ms/TE:40ms, matrix  = 64×64, voxel size 3×3×3mm, FoV 1152*1152). The sessions were randomized across the participants. In each session, the participants received 8 scans during the 20s-ON-block and 8 scans during the 20s-OFF-block according to Croy et al. [50]. ON and OFF blocks were repeated six times, each session lasting about 4 minutes. Additionally, T1-weighted images were acquired by using a 3D IR/GR sequence (TR: 2180ms/TE: 3.93ms; FoV 256*280/352*384) to localize the activated areas. The data analysis was performed with SPM 5 software (Statistical Parametric Mapping; Wellcome Department of Imaging Neuroscience, London, UK; www.fil.ion.ucl.ac.uk/spm), implemented in Matlab 7.9.0 (R2009b, The Mathworks Inc., USA) following spatial pre-processing with the same software (spatial filtering: high pass filter 128Hz, realignment, normalisation using a standard EPI template, smoothing by means of 8×8×8 FWHM). There were no significant group differences according to translational and rotational movement parameters [translation in mm: T = −.545, df = 24, p = .591, patients/controls:.58 (.42)/.49 (.36); rotation in °: T = .941, df = 24,.941, patients/controls:.08 (.60)/.06 (.80)]. The first three EPI images were discarded to allow the MRI signal to reach a steady state. MNI-Coordinates of the activation are presented. The localization of MNI-coordinates was realized with the Anatomy-Toolbox [51] and was confirmed with WFU-PickAtlas 2.4 [52] as well as the Talairach-Client [53]. The analysis was based on one-sample and two-sample t-tests. Voxels in MNI-space were considered statistically significant at a threshold of p<.05 (corrected for multiple comparisons at cluster level) using a height threshold of p<.001 uncorrected [50], corresponding to T = 3.26 and a cluster size of at least 10 activated voxels according to a recommendation by Lieberman and Cunningham [54]. In order to test our hypothesis of an altered activation in the olfactory processing areas, we performed region of interest (ROI) analyses with Small Volume Correction using masks for primary (amygdalae, piriform cortex) and secondary (orbitofrontal cortex, insula, hippocampus, thalamus) olfactory areas according to Sobel et al. [33] and Zatorre and Jones-Gotman [34]. The masks were created using the WFU PickAtlas 2.4 software [52]. Moreover, a hypothesis-driven ROI-analysis was performed according to the coordinates reported by Pillay et al. [25] for the anterior cingulate cortex [−2 16 −4] and Wittmann et al. [30] for the inferior frontal gyrus [45 30 −15] using a sphere centered at these coordinates with a radius of 10 mm. The BOLD-signal was correlated with PAS scores using Pearsońs correlation analysis. For the correlation analysis, we extracted the beta values (intensity) for the BOLD-contrasts which were of interest (ergometry > TSST for patients > controls for the cingulate gyrus [−4 −42 54] and TSST > ergometry for patients > controls for the inferior frontal gyrus [42 36 −10]). For each subject, the beta value was imported in SPSS version 21.0.0.0.

## Results

### Odor Ratings

**Figure 1:**
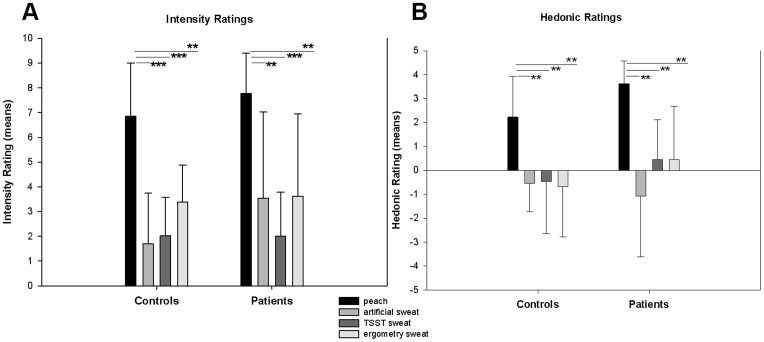
Intensity and hedonic ratings. Displayed are mean values and one-sided standard deviations for artificial odors (peach, artificial sweat) and own body odors (TSST sweat, ergometry sweat) in patients with PD and healthy controls. Intensity Ratings: 0 (no odor) − 10 (very strong intensity); Hedonic rating: −5 (very unpleasant) − +5 (very pleasant). ***p ≤.001, **p ≤.01

The odor ratings were analysed using the non-parametric Mann-Whitney U Test and the Wilcoxon signed-rank test. P-value was Bonferroni-corrected for multiple comparisons. There was no group difference in the perceived intensity of the odors (Mann-Whitney U Test-peach: U = 56.500, p = .146; artificial sweat: U = 60.000, p = .199; ergometry sweat: U = 76.500, p = .678; TSST sweat: U = 55.000, p = .121). Independent of the group, the peach odor was perceived as more intensive than the other odors (Wilcoxon signed-rank test for patients compared with artificial sweat: Z = −2.908, p = .004; ergometry sweat: Z = −2.979, p = .003; TSST sweat: Z = −3.219, p = .001; for healthy controls compared with artificial sweat: Z = −3.192, p = .001; ergometry sweat: Z = −2.914, p = .004; TSST sweat: Z = −3.195, p = .001). The other odors did not differ significantly with respect to the perceived intensity in both groups (Wilcoxon signed-rank test for patients- ergometry sweat vs. artificial sweat: Z = −.051, p = .959; TSST sweat vs. artificial sweat: Z = −.447, p = .655; TSST sweat vs. ergometry sweat: Z = −1.799, p = .072; for healthy controls- ergometry sweat vs. artificial sweat: Z = −2.176, p >.01; TSST sweat vs. artificial sweat: Z = −2.279, p >.01; TSST sweat vs. ergometry sweat: Z = −.709, p = .478) (see Figure 1).

According to the judged valence of the odors, there was no significant group difference between the healthy controls and the patients with PD (Mann-Whitney U Test- peach: U = 38.000, p >.01; artificial sweat: U = 83.000, p = .928; ergometry sweat: U = 57.500, p = .161; TSST sweat: U = 54.000, p = .110). There were no significant differences between the TSST sweat odor, the ergometry sweat odor, and the artificial sweat odor in both groups (Wilcoxon signed-rank test for patients- ergometry sweat vs. artificial sweat: Z = −1.535, p = .125; TSST sweat vs. artificial sweat: Z = −1.253, p = .210; TSST sweat vs. ergometry sweat: Z = −.212, p = .832; for healthy controls- ergometry sweat vs. artificial sweat: Z = −.278, p = .781; TSST sweat vs. artificial sweat: Z = −.040, p = .968; TSST sweat vs. ergometry sweat: Z = −.361, p = .718).

The sweat odors were rated as neutral to mildly unpleasant while the peach odor was rated as significantly more pleasant than the other odors (Wilcoxon signed-rank test for patients compared with artificial sweat: Z = −3.083, p = .002; ergometry sweat: Z = −2.943, p = .003; TSST sweat: Z = −2.931, p = .003; for healthy controls compared with artificial sweat: Z = −2.901, p = .004; ergometry sweat: Z = −2.594, p = .009; TSST sweat: Z = −2.728, p = .006) (see Figure 1).

### Main Effect of ‘‘artificial odors’’

First, we analyzed the main contrast ON- vs. OFF for artificial odors (artificial sweat, peach) separately for both groups, focusing on the aspect of olfactory processing. While the healthy controls activated both primary and secondary olfactory areas, patients with PD predominantly activated frontal areas not part of primary or secondary olfactory processing areas (for details see Table 2).

**Table 2.Relative:** increases in brain activity under the presentation of artificial odors.

Region		x	y	z	T(Z)-Score	kE (voxels)
**artificial odors > no odor, patients with PD**						
**Frontal Lobe**	_Inferior Frontal Gyrus_	_−50_	_30_	_16_	_5.85 (4.60)_	_706_
	_Middle Frontal Gyrus_	_−50_	_22_	_34_	_5.66 (4.50)_	_#_
	_Precentral Gyrus_	_−42_	_6_	_46_	_4.30 (3.68)_	_#_
	_Superior Medial Gyrus_	_−2_	_28_	_42_	_5.66 (4.50)_	_94_
	_Supplementary Motor area (SMA)_	_−10_	_20_	_46_	_3.74 (3.30)_	_#_
**Primary olfactory areas^1^**	_— no supratreshold voxels —_					
**Secondary olfactory areas^1^**	_— no supratreshold voxels —_					
**artifical odors > no odor, healthy controls**						
**Corpus Callosum**		_0_	_6_	_16_	_7.01 (5.16)_	_1199_
		_−6_	_−6_	_22_	_6.82 (5.08)_	_#_
		_6_	_−4_	_20_	_6.30 (4.83)_	_#_
**Sublobular**	_Nucleus Caudatus_	_18_	_16_	_18_	_5.42 (4.37)_	_#_
**Limbic Lobe**	_Cingulate Gyrus_	_−6_	_−6_	_22_	_6.82 (5.08)_	_#_
	_Parahippocampal Gyrus_	_10_	_−36_	_0_	_4.40 (3.75)_	_#_
	_Mammillary Body_	_4_	_−6_	_−12_	_3.98 (3.47)_	_100_
**Frontal Lobe**	_Inferior Frontal Gyrus_	_−44_	_42_	_12_	_6.38 (4.87)_	_201_
	_Middle Frontal Gyrus_	_−42_	_46_	_24_	_4.03 (3.50)_	_#_
**Temporal Lobe**	_Inferior Temporal Gyrus_	_60_	_−42_	_−10_	_4.55 (3.85)_	_106_
	_Middle Temporal Gyrus_	_64_	_−42_	_−14_	_4.41 (3.76)_	_#_
**Parietal Lobe**	_Precuneus_	_−24_	_−52_	_10_	_4.84 (4.03)_	_89_
**Calcarine Gyrus**		_−26_	_−60_	_10_	_4.49 (3.81)_	_#_
**Primary olfactory areas**	_Amygdala_^1^	_18_	_−6_	_−20_	_4.59 (3.87)_	_28_
		_18_	_−8_	_−16_	_4.44 (3.77)_	_#_
**Secondary olfactory areas**	_Insula_^1^	_−42_	_−16_	_10_	_4.59 (3.88)_	_37_
		_−46_	_−10_	_4_	_3.89 (3.41)_	_#_
	_Orbitofrontal Cortex_^1^	_46_	_54_	_0_	_5.18 (4.23)_	_35_
		_46_	_38_	_−14_	_5.03 (4.14)_	_30_
**artificial odors > no odor, patients with PD > healthy controls**						
	_— no supratreshold voxels —_					
**artificial odors > no odor, healthy controls > patients with PD**						
	_Thalamus_	_8_	_−34_	_2_	_4.19 (3.86)_	_121_
		_8_	_−28_	_4_	_4.17 (3.85)_	_#_
**Limbic Lobe**	_Posterior Cingulate_	_10_	_−38_	_8_	_3.96 (3.67)_	_#_
	_Anterior Cingulate_^2^	_0_	_22_	_−8_	_3.95 (3.67)_	_35_
**Primary olfactory areas^1^**	_— no supratreshold voxels —_					
**Secondary olfactory areas^1^**	_Thalamus_	_8_	_−28_	_4_	_4.17 (3.85)_	_25_
		_6_	_−26_	_8_	_4.03 (3.73)_	_#_

Brain activation for the contrast artificial odors > no odor for the pooled odors peach and artificial sweat for patients with PD (N = 13), controls (N = 13) and for patients > controls/controls > patients. Whole brain analyses are corrected at cluster level and uncorrected at a height threshold of p<.001.

All activations are significant at p<.05, corrected for multiple comparisons at the cluster level (with a height threshold of p<.001, uncorrected). 1 = p<.05 in a hypothesis-driven region-of-interest (ROI) analysis. Brain masks were created using WFU PickAtlas. 2 = p<.05 in a hypothesis-driven region-of-interest (ROI) analysis according to the coordinates reported by by Pillay et al. [25] for the anterior cingulate cortex [−2 16 −4].

# indicates that this activation maximum is part of the same cluster.

For each region of activation, the coordinates of the maximally activated voxels within the activation cluster are given in standard stereotactic MNI space.

### Comparison between the groups for “artificial odors”

We compared the odor - no odor contrasts for artificial odors (peach, artificial sweat) of the patients with PD with the odor - no odor contrasts of the healthy controls (odor > no odor; PD >/<controls) and performed a Small Volume Correction for coordinates of the anterior cingulate cortex [−2 16 −4] reported by Pillay et al. in patients with PD [25].

**Figure 2:**
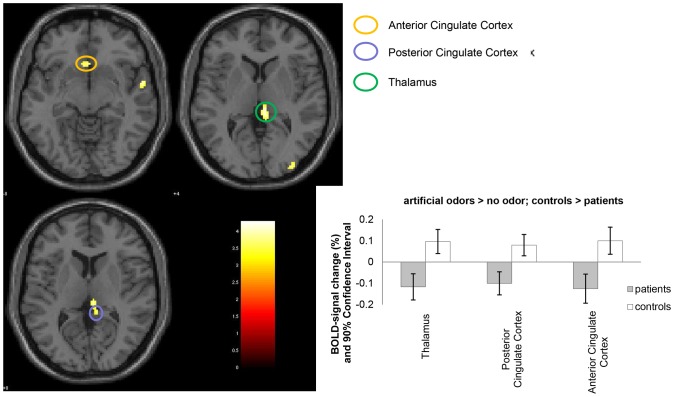
Activated clusters for the contrast artificial odors (peach, artificial sweat) > no odor. The contrast is displayed for the healthy controls compared to patients with PD (K≥10, p<.05). For visualization a normalized template provided by SPM5-Software (single_subj_T1.nii) was used. *p<.05 in a hypothesis-driven region-of-interest (ROI) analysis according to the coordinates reported by Pillay et al. [25] for the anterior cingulate cortex [−2 16 −4].

The contrast revealed no suprathreshold activations of olfactory processing areas in the group of patients with PD compared to the healthy controls. However, the controls showed an increased activation in the right thalamus, the right posterior cingulate cortex, and the left anterior cingulate cortex compared to the patients with PD (for details see Table 2 and Figure 2). The comparison of both the odor - no odor contrasts during the presentation of the artificial sweat odor and the peach odor did not reveal any significant group differences.

### Main Effect of ‘‘own body odors’’

Second, we analyzed the odor - no odor contrasts for own body odors (anxiety sweat, ergometry sweat) separately for both groups. While the patients with PD activated frontal and temporal areas (see Table 3), the healthy controls activated parts of the limbic lobe with the posterior and the anterior cingulate cortex (see Table 3). In ROI-analyses, both groups did not show a significantly enhanced activation in areas that are typically involved in the processing of olfactory stimuli such as the primary olfactory cortex (e.g. piriform cortex, amygdalae, entorhinal cortex) or the secondary olfactory cortex (e.g. thalamus, hypothalamus, hippocampus, insula, orbitofrontal cortex). Only the patients with PD showed enhanced activation of the orbitofrontal cortex as part of the secondary olfactory area.

**Table 3:** Relative increases in brain activity under the presentation of own body odors.

Region		x	y	z	T(Z)-Score	kE (voxels)
**own body odors > no odor, patients with PD**						
**Frontal Lobe**	_Inferior Frontal Gyrus_	_−48_	_14_	_0_	_5.38 (4.35)_	_63_
**Temporal Lobe**	_Superior Temporal Gyrus_	_48_	_−10_	_0_	_4.03 (3.51)_	_57_
**Primary olfactory areas^1^**	_— no supratreshold voxels —_					
**Secondary olfactory areas^1^**	_Orbitofrontal Cortex*_	_−48_	_16_	_0_	_4.69 (3.93)_	_12_
**own body odors > no odor, healthy controls**						
**Temporal Lobe**	_Superior Temporal_ _Gyrus_	_32_	_−46_	_10_	_5.05 (4.16)_	_93_
**Limbic Lobe**	_Cingulate Gyrus_	_−18_	_−38_	_16_	_4.08 (3.54)_	_56_
	_Posterior Cingulate_	_−10_	_−30_	_22_	_4.41 (3.76)_	_47_
	_Cingulate Gyrus_	_−12_	_−22_	_24_	_4.09 (3.54)_	_#_
**Primary olfactory areas^1^**	_— no supratreshold voxels —_					
**Secondary olfactory areas^1^**	_— no supratreshold voxels —_					
**ergometry > no odor, patients with PD > healthy controls**						
**Temporal Lobe**	_Superior Temporal Gyrus_	_52_	**_−_**_32_	_16_	_4.31 (3.67)_	_40_
**Parietal Lobe**	_Supramarginal Gyrus_	_52_	**_−_**_30_	_24_	_4.17 (3.58)_	_#_
**TSST > no odor, patients with PD > healthy controls**						
	_— no supratreshold voxels —_					
**ergometry > TSST, patients with PD > healthy controls**						
**Limbic Lobe**	_Cingulate Cortex_	_−4_	_−42_	_54_	_4.51 (3.80)_	_41_
**Parietal Lobe**	_Supramarginal Gyrus_	_48_	_−26_	_34_	_4.58 (3.84)_	_40_
**TSST > ergometry, patients with PD > healthy controls**						
**Frontal Lobe**	_Inferior Frontal Gyrus_^2^	_42_	_36_	_−10_	_5.14 (4.18)_	_21_

Brain activation for the contrast own body odors > no odor for the pooled odors TSST sweat and ergometry sweat for patients with PD (N = 13), controls (N = 13) and for patients > controls/controls > patients. Whole brain analyses are corrected at cluster level and uncorrected at a height threshold of p<.001.

All activations are significant at p<.05, corrected for multiple comparisons at the cluster level (with a height threshold of p<.001, uncorrected).

1 = p<.05 in a hypothesis-driven region-of-interest (ROI) analysis. Brain masks were created using WFU PickAtlas. 2 = p<.05 in a hypothesis-driven region-of-interest (ROI) analysis according to the coordinates reported by Wittmann et al. [30] for the inferior frontal gyrus [45 30 −15].

# indicates that this activation maximum is part of the same cluster.

For each region of activation, the coordinates of the maximally activated voxels within the activation cluster are given in standard stereotactic MNI space.

### Comparison between the groups for “own body odors”

The comparison of the odor - no odor contrasts during the presentation of ergometry sweat revealed more brain activation in the superior temporal gyrus and the supramarginal gyrus in the patients with PD than in the healthy controls (see Table 3). There were no significant group differences when the odor-no odor contrasts during the presentation of anxiety sweat were compared.

**Figure 3:**
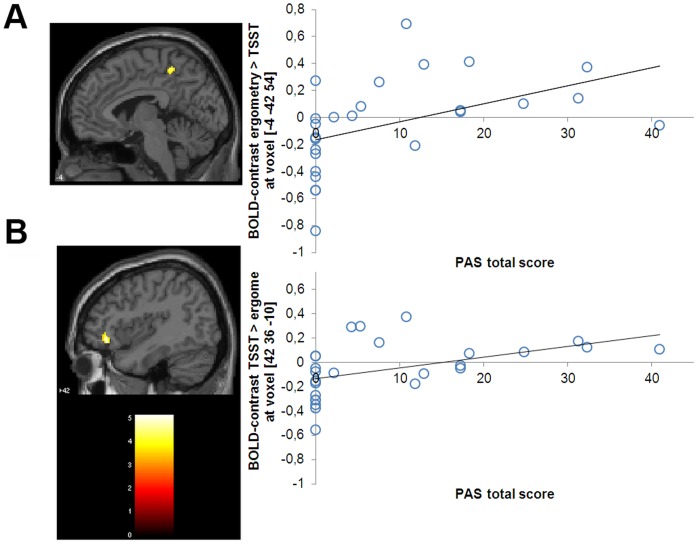
Activated cluster for the contrast ergometry sweat odor > TSST sweat odor (Fig. 3a). The contrast is displayed for the comparison of patients with PD and healthy controls (K≥10, p<.05). Patients showed more activation in the left cingulate cortex than healthy controls. Activated cluster for the contrast TSST sweat odor > ergometry sweat odor (Fig. 3b). Patients showed more activation in the right inferior frontal gyrus than healthy controls. The activated clusters were significantly positive correlated with the severity of psychopathology on the PAS [41]. For visualization, we used a normalized template, provided by SPM 5- Software (single_subj_T1.nii).

When the two sweat conditions were compared between groups, the patients showed more brain activation under the presentation of the ergometry sweat odor compared to the anxiety sweat odor in the cingulate cortex and the supramarginal gyrus. The activation in the left cingulate cortex was positively correlated with the total score in the Panic- and Agoraphobia-Scale (PAS) (Pearsońs r  = .465, p = .017, see Figure 3a). Moreover, correlations were found with the subscales of the PAS: agoraphobic avoidance (Pearson`s r  = .540, p = .004), anticipatory anxiety (Pearsońs r  = .488, p = .011) and health concerns (Pearsońs r  = .406, p = .040).

The comparison of the odor - no odor contrast during the presentation of the anxiety sweat odor did not reveal any significant group differences. In a ROI-analysis according to the MNI-coordinates [42 36 −10] reported by Wittmann et al. [30], patients with PD showed more brain activity in the inferior frontal gyrus under the presentation of anxiety sweat odor compared to ergometry sweat odor. This was positively correlated with the total score in the Panic- and Agoraphobia-Scale (PAS) (Pearsońs r  = .462, p = .017, see Figure 3b). Moreover, correlations were found with the subscales of the PAS: agoraphobic avoidance (Pearson`s r  = .493, p = .010), anticipatory anxiety (Pearsońs r  = .442, p = .024) and health concerns (Pearsońs r  = .468, p = .016).

## Discussion

Recent findings showed an overlap between a panic-specific fear network, neuronal areas associated with emotional processing, and the olfactory projection areas [4], [5], [23], [24], [32]. Therefore, the present study focused on the neuronal processing of odors gained under potential psychosocial threat and under physical exercise in patients with PD. It was hypothesized that the presentation of odors gained during potentially threatening situations might trigger a panic-specific fear-network in patients with PD [4].

Under the presentation of **own body odors** (TSST sweat, ergometry sweat), patients with PD showed activations in inferior frontal, superior temporal, and orbitofrontal areas while healthy controls also activated cerebellum and limbic lobe structures such as the cingulate and the posterior cingulate cortex.

When the two sweat conditions were compared between the groups, the patients showed more brain activity under the presentation of the ergometry sweat odor compared to the TSST sweat odor in the cingulate gyrus and supramarginal gyrus. An increased activation in the cingulate cortex was accompanied by an increased total score of the Panic- and Agoraphobia-Scale, increased agoraphobic avoidance, anticipatory anxiety, and health concerns. Under the presentation of the TSST sweat odor compared to the ergometry sweat odor, the patients with PD showed more brain activity in the inferior frontal gyrus. The latter was also positively correlated with the total score in the Panic- and Agoraphobia-Scale, agoraphobic avoidance, anticipatory anxiety, and health concerns.

The patients with PD **predominantly activated inferior frontal parts** rather than the amygdalae under the presentation of their **own body odors**. This is in line with the literature showing lacking activation of the amygdalae even under the presentation of panic-specific stimuli e.g. panic-related words, fearful faces, or panicogenic agents [fMRI studies: e.g. 23,24,25,26,55]. This lacking activation of limbic areas has also been shown in patients with schizophrenia who rather showed an extensive ventral, medial, and dorsolateral frontal activation while failing to activate limbic and paralimbic regions under the presentation of an unpleasant odor stimulus [56]. Crespo-Facorro and colleagues concluded that the enhanced activation of prefrontal brain regions might be compensatory for the failure of the limbic/paralimbic regions to distinguish unpleasant from pleasant or neutral stimuli [56]. This activation pattern might also occur in patients with PD and might be accompanied by the panic-specific overestimation of threat and negative consequences as well as a pre-attentive processing of negative stimuli [1], [5], [56]. In addition, a lacking activation of the amygdalae under the presentation of stress-related sweat odors in patients with PD might also be due to the fact that in the present study moderately unpleasant olfactory stimuli and no specific panic-related odors such as odors gained during first-time sky diving were used [9], [30].

With respect to their own body odors, the patients with PD showed an enhanced activity predominantly in the inferior frontal gyrus. Based on the neuroanatomical model, activation in the medial prefrontal cortex is associated with anticipatory anxiety and avoidant behaviour [4]. The medial prefrontal cortex plays an important role for the information processing of onés own and otherś emotional states [57], the formation of dysfunctional interpretation of somatic symptoms, and agoraphobic avoidance [4]. Previous findings could show a correlation between the activation of the inferior prefrontal cortex with the severity of psychopathology [58], [59], [60], anticipatory anxiety [23], [30], [61], and avoidant behaviour in patients with PD [62]. Recent results from a differential conditioning paradigm were able to show an increased activation of the inferior frontal gyrus during exteroceptive fear conditioning [31]. The authors conclude that an increased activity of the inferior frontal gyrus is related to enhanced top-down control when risk assessment and threat evaluation take place. In the present study, an enhanced activity of the inferior and orbitofrontal cortex could be shown for the presentation of own body odors. Also, own body odors from stressful situations might display potentially threatening stimuli, increasing the necessity to enhance top-down control and behavioural inhibition in order to show an adaptive behavioural response [31], [63].

In addition, the presentation of **artificial odors** led to a **reduced activation of the anterior cingulate cortex and the posterior cingulate cortex** in the patients with PD. Recent findings from a previous study showed a reduced activation of the anterior cingulate cortex under the presentation of neutral (coffee) to very pleasant (peach) odors in patients with childhood maltreatment [50]. In patients with PD, a reduced activation of both the amgydalae and the anterior cingulate cortex could be observed under the presentation of fearful stimuli (faces) [25]. Even under remitted psychopathology, a reduced activation of the anterior cingulate cortex could be found for not-panic-specific word-face pairs in patients with PD [27]. The anterior and posterior cingulate cortex play an important role for selective attention [64], emotional evaluation, and modulation [64], [65]. Patients with PD show selective attention towards threat [66] and enhanced recruitment of episodic memory structures located in the posterior cingulate cortex [24], [67], which is activated in a sustained way during baseline-conditions and thus deactivated under stimulation [68]. Furthermore, patients with PD reduce their attentional resources when faced with a threatening situation as an adaption strategy based on their chronic hyper-arousal in order to prevent a possible somatic/neuronal over-reaction. This might be reflected in a reduced activity of the anterior and posterior cingulate cortex [25].

Thus, one might conclude that the artificial odors led to an enhanced arousal accompanied by a modulation of attentional resources away from a subsequent potential threat to maintain their ability to react and not to be overwhelmed [25].

In the present study, we found **reduced activity of the thalamus** under the presentation of **artificial odors.** The thalamus is a major part of the fear-network [4] which regulates the attentional processes, memory, and speech by involving the efferents from the amygdalae to the thalamus [69]. The thalamus has a pivotal role in the coordination of neocortical attentional control systems and the maintenance of attention. A disturbed neuronal transmission of sensory information from the thalamus to the amygdalae, the anterior cingulate cortex, and the hippocampus might therefore increase the susceptibility to experiencing anxiety symptoms [70]. These processes might be altered under the presentation of olfactory stimuli in patients with PD.

The strengths of the present study are the use of a structured clinical diagnostic interview (SCID, [36], [37]) for the assessment of PD and the inclusion of patients without other psychiatric comorbidities as well as psychotropic drug treatment or medication with impact on the olfactory system. Healthy controls were matched by age and gender. A block design was used which allows a large BOLD- signal change [71].

Our results should be interpreted within the context of methodological limitations. In the present study, approximately two thirds of the patients with PD had a remitted to mild severity of PD. The mild symptom severity might be responsible for the findings that specific neuronal areas such as the amgydalae (which is usually activated to trigger flight or fight behaviour in PD), do no longer show any enhanced activation [72]. Therefore, future studies should include a group of patients with more severe psychopathology. Moreover, future studies should also include a patient control group in order to investigate whether the neuronal alterations in olfactory processing are specific to patients with PD. In order to exclude alterations in the breathing regime, future studies should control breathing by way of a chest belt and supply support via computerized visual cues clearly indicating inhalation and exhalation phases [9], [20].

Brain activity data were analyzed using a rather flexible height threshold of p<.001 (uncorrected) with a correction for multiple comparisons at cluster level. Although this approach is in line with a former study that used a similar kind of low-potent odor stimuli [50], future studies could apply more conservative thresholding in a larger sample. As another limitation, patients with PD did not show activations of primary and secondary olfactory processing areas. In general, due to the small sample size, the group differences may not have been detected and, hence, the data should be interpreted with caution. The findings should be replicated in a larger sample of patients with PD. In order to elicit the amygdalae activation, future studies might apply more panic-specific and more potent body odor stimuli such as sweat odor generated during a first-time tandem skydive [9].

In summary, our findings suggest some differences in the neuronal processing of stress-related body-odors and artificial odors in patients with PD. These differences in the neural activity might be associated with an increased severity of the psychopathology and dysfunctional threat-related cognitive processing. The differences in olfactory processing might display a vulnerability factor in patients with PD, which might even be sustained in patients with a mild or remitted psychopathology.
