# Correction: Altered Olfactory Processing of Stress-Related Body Odors and Artificial Odors in Patients with Panic Disorder

**DOI:** 10.1371/annotation/e2406113-a289-49f6-b3d9-88d09d385d50

**Published:** 2013-10-15

**Authors:** Gloria-Beatrice Wintermann, Markus Donix, Peter Joraschky, Johannes Gerber, Katja Petrowski

A funding organization was incorrectly omitted from the Funding Statement. The following sentence should be included with the Funding Statement: "We would like to thank the Robert-Pfleger-Foundation for giving a fund to support the present study." 

